# Detection of autoantibodies against heat shock proteins and collapsin response mediator proteins in autoimmune retinopathy

**DOI:** 10.1186/1471-2415-13-48

**Published:** 2013-09-25

**Authors:** Grazyna Adamus, Robert Bonnah, Lori Brown, Larry David

**Affiliations:** 1 Ocular Immunology Laboratory, Oregon Health and Science University, 3181 SW Sam Jackson Pk Rd, Portland, OR 97239, USA; 2 Casey Eye Institute, and Department of Biochemistry & Molecular Biology, Oregon Health and Science University, Portland, OR, USA; 3 Current address: Oregon Stem Cell Center, Oregon Health and Science University, 3181 SW Sam Jackson Pk Rd, Portland, OR 97239, USA

**Keywords:** Autoantibody, Autoimmune retinopathy, CAR, Retina, Autoantigen, Heat shock proteins, CRMP-2

## Abstract

**Background:**

Autoimmune retinopathy (AR) and Cancer-Associated Retinopathy (CAR) are associated with a diverse repertoire of anti-retinal autoantibodies (AAbs) but not all antigenic targets have been characterized. Identification of new AAbs may help with clinical diagnosis and prognosis of retinal dysfunction in AR. The goal was to identify frequently targeted retinal autoantigens within the 60-70-kDa molecular weight range.

**Methods:**

Human retinal proteins were separated by SDS-PAGE and 2D gel electrophoresis (2-DE) and sera from AR patients with and without cancer were used to identify immunoreactive proteins by Western blotting. Proteins were identified following separation by electrophoresis, Coomassie staining using in-gel trypsin digestion and mass spectrometric analysis. Circulating serum hsp60 and anti-hsp60 antibody levels were determined by quantitative ELISA.

**Results:**

Retrospective evaluation of 819 patients with anti-retinal AAbs showed that 29% patients had AAbs targeted proteins between 60-70-kDa. Shotgun mass spectrometry of human retinal proteins present in 1D-gel found 66 species within this range. To identify the immunoreactive proteins, we performed Western blots of 2-DE gels and showed a group of heat shock proteins (hsps), including hsp60 and CRMP proteins that were frequently recognized by AR patient AAbs, irrespective of cancer status. These results were validated by immunostaining of purified hsp60 and CRMP2 proteins. ELISA results revealed that patients with AR and CAR had significantly increased levels of serum anti-hsp60 antibodies compared to control healthy subjects (p < 0.0001). However, circulating hsp60 protein was not significantly elevated in sera of either patient group.

**Conclusions:**

Different anti-retinal antibodies frequently co-exist in a single patient, creating antibody-arrays related to the syndrome. Hsps and CRMP-2 are newly identified autoantigens in AR. A frequent co-association of anti-hsp antibodies with other anti-retinal AAbs may augment pathogenic processes, leading to retinal degeneration.

## Background

Retinal degeneration is one of the most common forms of untreatable blindness and can result from many causes, both genetic and acquired. Autoimmunity is increasingly recognized as possible underlying cause of retinal degeneration in autoimmune retinopathies (AR), including paraneoplastic syndromes, such as cancer-associated retinopathy (CAR) and melanoma-associated retinopathy (MAR). However, our understanding of the pathogenicity of autoimmune retinopathies is still incomplete. Evidence suggests that autoantibodies (AAbs) specific to distinct retinal antigens are present in sera from patients with AR. AR affects middle age people over 50 years old and is characterized by the sudden onset and progressive loss of vision, photopsia, visual field loss, and abnormal ERG findings [1]. AR occurs in some patients who are also predisposed to autoimmune diseases or have family history of autoimmune diseases [2]. Studies suggest that progression of retinal degeneration over time could result from the attack by the immune system on healthy retina [3-5].

As in other autoimmune disorders, AR including CAR is characterized by the presence of a diverse serum autoantibody repertoire, but not all target antigens have been identified [1,6]. Moreover, heterogeneity in anti-retinal autoantibody recognition may produce distinctive retinal disorders [4,7-9]. In fact, the profiling of AAbs led to valuable data aiding clinical diagnosis and prognosis of retinal dysfunction in AR; for instance, anti-recoverin AAbs are associated with severe retinal dysfunction of rods and cones whereas anti-enolase AAbs are associated with slow progressive dysfunction mostly in cones [7,8]. Anti-transducin phenotype is also characterized by defects in visual fields and reduced scotopic ERG responses [9]. It is also important to point out that in CAR, retinal dysfunction and AAbs may manifest months or years prior to the onset and diagnosis of cancer, thus could also be useful biomarkers for this disease [1]. Anti-retinal antibodies persist in the circulation and their levels fluctuate over long periods after the onset of visual symptoms [6,10]. We believe that identification of new AAbs will help to elucidate the pathophysiology in AR, and ultimately facilitate novel immune treatments, such as antigen-tolerating therapy. In this study, we identified heat shock protein 60 (hsp60) and collapsin response mediator protein 2 (CRMP2) as possible autoantigens frequently observed in patients with CAR, MAR and AR. Anti-Hsp60 autoantibodies that often co-existed with other anti-retinal AAbs, including anti-recoverin and anti-α-enolase, may contribute to the autoimmune pathology in AR.

## Methods

### Patient sera

Patients’ sera were acquired form the Serum Repository of Oregon Health & Science University (OHSU). The studies were approved by the OHSU Institutional Review Board and our research adhered to the tenets of the Declaration of Helsinki. The subjects presented unexplained, progressive visual loss, night vision loss, defects in visual field –paracentral or central scotoma, abnormal ERG, and had either cancer or the suspicion of cancer. We retrospectively evaluated 1260 retinopathy patients’ western blotting data and identified 237 patients with AAbs against retinal proteins of an apparent molecular range of 60-70-kDa.

### Western blotting

Human retinal proteins were extracted from a donor retina with 2% octyl glucoside in phosphate buffer (pH 7.2) and 10 μg of protein per lane was separated by SDS-gel electrophoresis using 10% Bio-Rad Criterion gels (Hercules, CA, USA) followed by transfer to a PVDF membrane as described before [6]. After two-dimensional (2-D) gel electrophoresis (see below) retinal proteins were also transferred to the PVDF membrane. Blots were then immunostained with 1:25 diluted patient sera followed by incubation with anti-human IgG (H and L chain) conjugated to alkaline phosphatase (Invitrogen, Grand Island, NY, USA). The immune reaction was then developed using AP substrate (Invitrogen). As positive controls in 1-D blots, we used a reference human serum containing anti-enolase AAbs at 1:100, anti-enolase Enol-1 MAb at 1:2000, and anti-recoverin R2 antiserum at 1:50,000 dilutions (both antibodies were developed in our laboratory). A negative control contained secondary antibodies only.

For verification experiments we used purified human recombinant Hsp60 and recombinant M. bovis Hsp65 (native) protein purchased from StressMarq Bioscience (Victoria, BC, Canada), and the appropriate secondary antibodies against human Hsp60, Hsp65, and Hsp70 from Cell Signaling Technology (Danvers, MA, U.S.A.). Collapsin response mediator protein 2 (CRMP-2; Kinasource, UK) was used for western blots using the control sheep anti-human CRMP-2 antibodies form Kinasource, UK.

### Immunomics - a library of 60-70-kDa retinal antigens

This methodology was to identify retinal proteins reactive with patient AAbs within a 60-70-kDa molecular range and consisted of 3 essential steps: (i) the preparation of retinal protein extracts, (ii) the screening of autoantigens by Western blotting, and (iii) the identification of the relevant proteins from the gel using mass spectrometry. The identification of protein antigens was performed as follows: 30 μg of human retinal proteins were separated by SDS-PAGE and then stained with Coomassie brilliant blue. The slabs of 1 D-gels within the desired molecular weight range (60-70-kDa) were cut into 1-mm pieces, and subjected to in-gel trypsin digestion as previously described [11]. Protein identification was performed by liquid chromatography-tandem mass spectrometry (LC-MS/MS) of peptides using Agilent 1100 series capillary LC system, 140 min long acetonitrile gradient and reverse phase column, and data-dependent MS/MS scans using an LTQ linear ion trap mass spectrometer (Thermo Scientific, San Jose, CA) [11]. The resulting MS/MS spectra were matched to theoretical fragmentation spectra of peptides generated from a human-only version of the Swiss-Prot database (Swiss Institute of Bioinformatics, Geneva, Switzerland) containing 19042 entries using Sequest (version 27, rev. 12, Thermo Scientific). A static mass shift of 57 was added to all cysteine residues to account for carbamidomethylation, and a differential mass shift of 16 was applied to methionine residues to detect possible oxidation. Parent and fragment ion tolerances of 3 and 1 Da were used, respectively, and no enzyme specificity was used during the search. The program Scaffold (version 3.6.5, Proteome Software Inc., Portland, OR) was used to validate MS/MS-based peptide and protein identifications. Minimum peptide and protein identification probabilities of 80 and 99% were required respectively, and a minimum of at least 2 unique peptide assignments to each identified protein.

### Two-dimensional gel electrophoresis and mass spectrometry

Human retinal extracts were desalted and 150 μg of proteins were loaded onto a rehydrated IPG strip (11 cm) overnight using pH range 5–8. The isoelectrofocusing was run at 30,000 volt hours and then the strips were stored at −80°C until use. The second dimension electrophoresis was run using 10% Bio-Rad Criterion gels in Tris-Glycine buffer, pH 8.3 at 150 V for 1 hr. The gels were stained in colloidal Coomassie Blue G250 and photographed, or transferred to a PVDF membrane for western blotting analysis. Protein spots were excised from the gel based on the antibody reactivity with antigens on 2-D blots and then identified by mass spectrometry as described above.

### Quantitative ELISA

Specific tests for determination of human Hsp60 proteins and anti-Hsp60 antibodies in serum were performed using commercial kits from Enzo Life Sciences (Plymouth, PA) following the manufacture’s procedures. We quantified circulating Hsp60 in 1:500 diluted sera from 20 AR patients and 20 normal patients in duplicate using a quantitative sandwich immunoassay. The second assay quantified of anti-Hsp60 antibodies in 1:500 diluted sera of 27 control patients and 46 AR patients. The color reaction was measured at 450 nm using a Bio-Rad Microplate Reader. Statistical analysis was performed with GraphPad Prism software (GraphPad Software, Inc. San Diego, CA) using a two-tailed Man Whitney *t* test. Differences with a p value < 0.05 were considered significant and are denoted by an asterisk.

## Results

**Figure 1:**
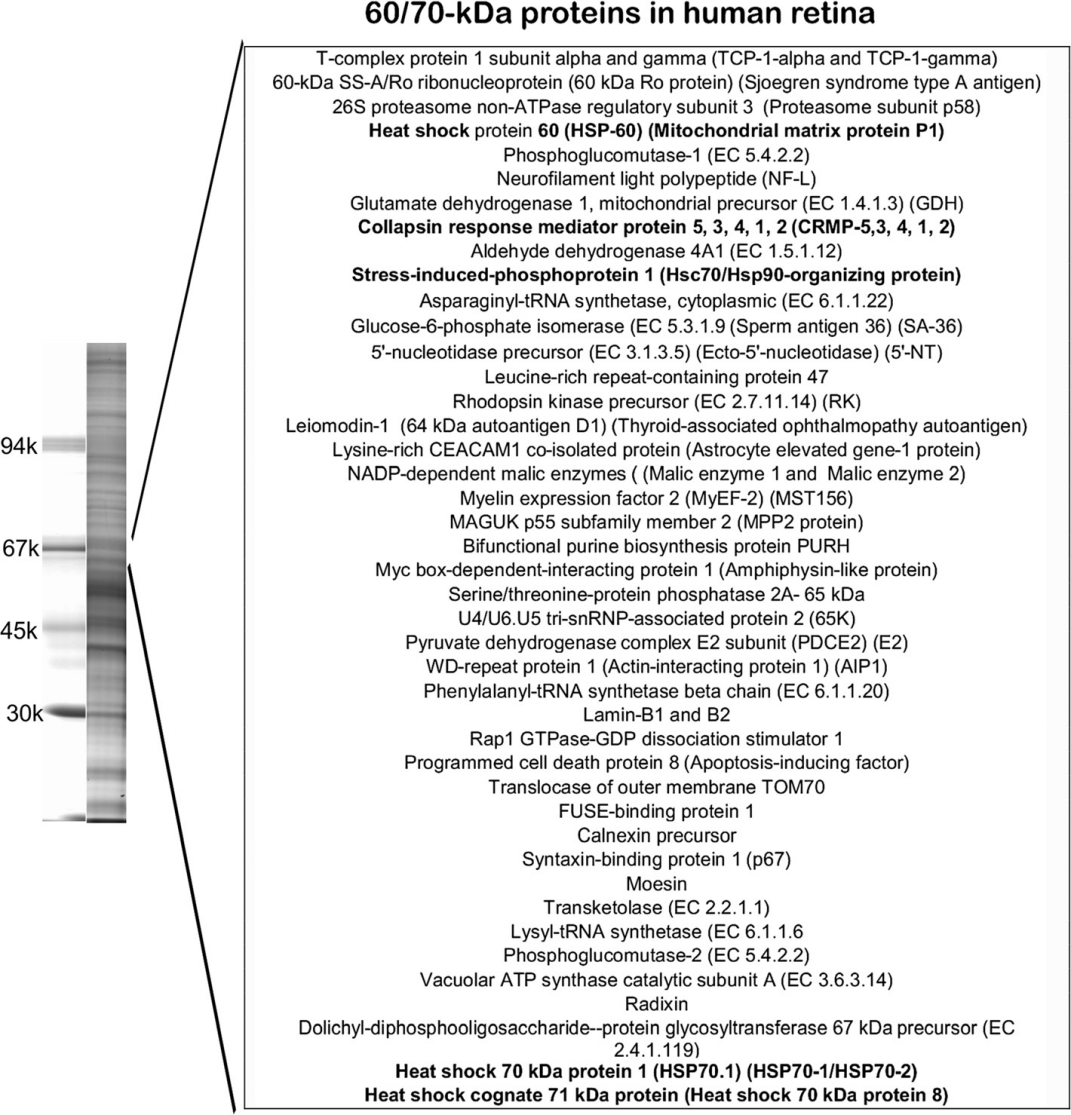
**Human retinal proteins found in the 1-D gel within the molecular weight 60 to 70-kDa range.** Proteins were identified using in-gel digestion and mass spectrometry (see Methods). On the left, a picture representing a separation of human retinal proteins on 10% gel.

We observed a diverse retinal antigen recognition pattern by human AAbs as tested by Western blotting and not all retinal proteins targeted by AAbs have been identified at the molecular level. Retrospective evaluation of 1260 sera from patients with ocular symptoms of AR tested for anti-retinal AAbs revealed 819 seropositive samples. Based on western blot analysis, about 29% of those seropositive patients had AAbs that targeted retinal proteins at a molecular range from 60-70-kDa presenting as single specificity AAbs in the serum or co-existed with other antibodies, including anti-recoverin or anti-enolase in those patients. In contrast, 15% of control subjects had AAbs reacting with 60-70-kDa retinal proteins. Proportionally, AAbs against a ~62-kDa protein were more common than against a 70-kDa protein. To identify potential retinal autoantigens, we first identified all major retinal proteins present in the 60–70 kDa range on a 1-D SDS-PAGE gel using in-gel digestion and mass spectrometry. This procedure identified 66 human retinal proteins within the desired molecular range (Figure 1) as candidate antigenic targets. Among the proteins from the gel we found a group of heat shock proteins (hsps), including heat shock protein 60 (CH60_HUMAN), heat shock 70 kDa protein 1A/1B (HSP71_HUMAN), heat shock 70 kDa protein 12A (HS12A_HUMAN), and heat shock cognate 71 kDa protein (HSP7C_HUMAN).

**Figure 2:**
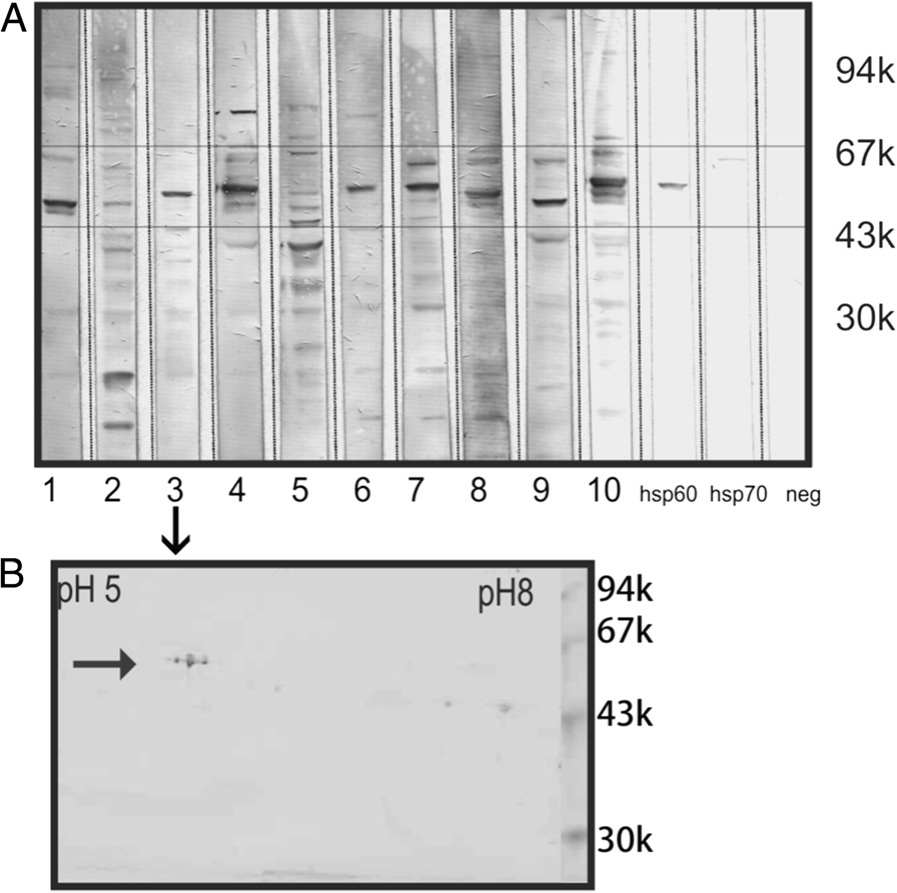
**Western blotting of human retinal proteins with 10 representative sera of autoimmune retinopathy patients. (A)** Immunoblotting shows that autoantibodies bind to different retinal proteins within the molecular range of 60 to 70-kDa (boxed). These sera 1–10 were selected to be further analyzed by 2-D western blotting (see Figure 3). **(B)** A representative 2-D western blot incubated with serum#3 shows 3 molecular forms of 62-kDa protein. Negative control – no primary antibody, positive controls – specific antibodies against human Hsp60 and Hsp70 proteins.

**Figure 3:**
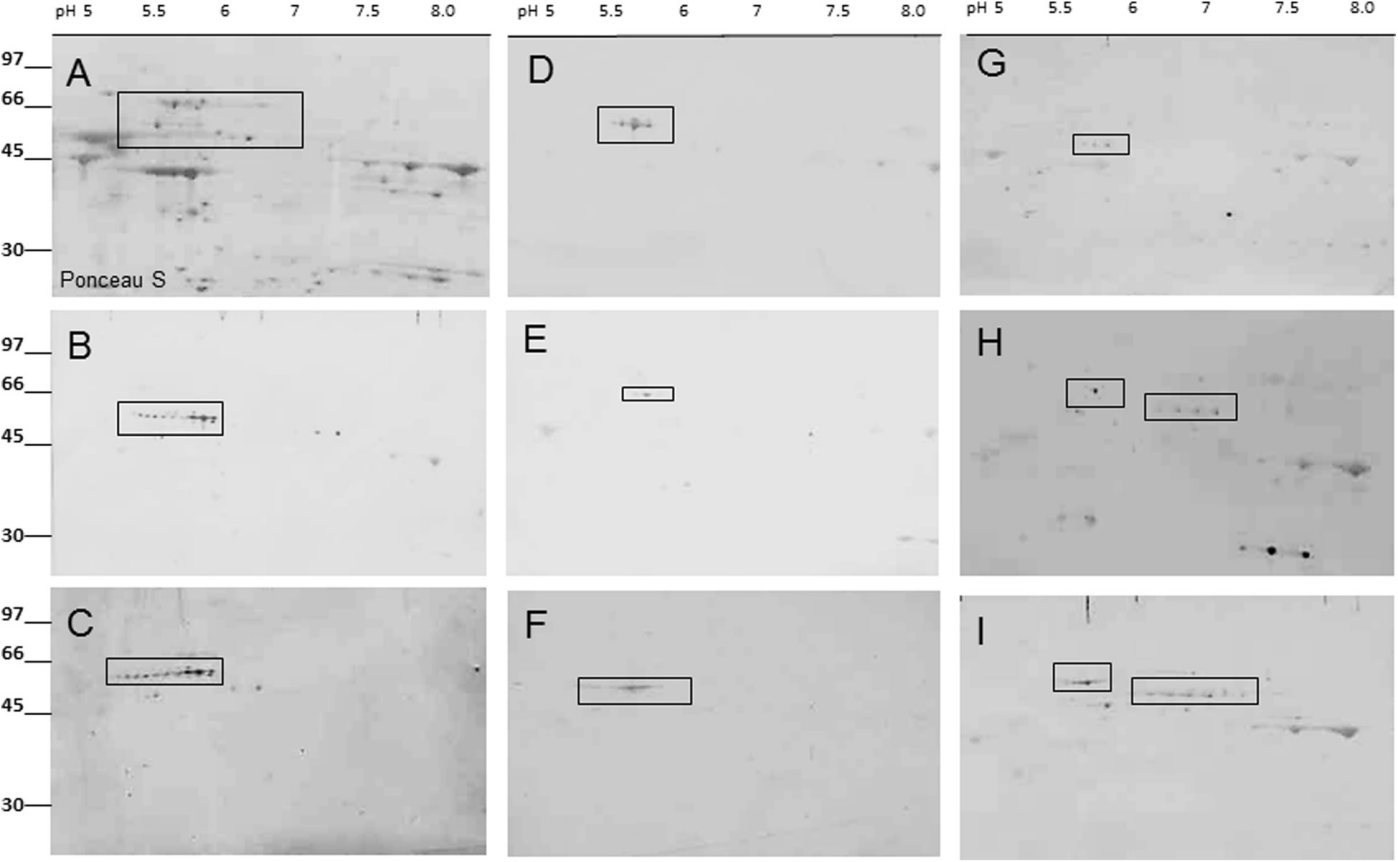
**Representative 2-D western blot analysis of patients sera analyzed by 2-dimensional SDS-PAGE of human retinal proteins; Proteins were first separated by 2-D gel electrophoresis and transferred to a PVDF membrane, then the membrane was stained Ponceau S (A) and destained before incubation with human sera at 1:100 dilution overnight (B-I).** Molecular standards are shown on the left and pH range is shown on the top of 2-D gel. The boxed spots on the blots marked the positions of excised proteins from the companion CBB stained gel that was analyzed by mass spectrometry. In **B**, **C**, **D**, and **E** - sera contained AAbs against hsp60, in **F** – serum with AAbs against hsp70, and in **G**, **H**, **I** - sera contain mainly AAbs against CRMP-2 and 3. Multiple spot of nearly horizontal spots of the same protein represent isoforms of the same protein.

**Figure 4:**
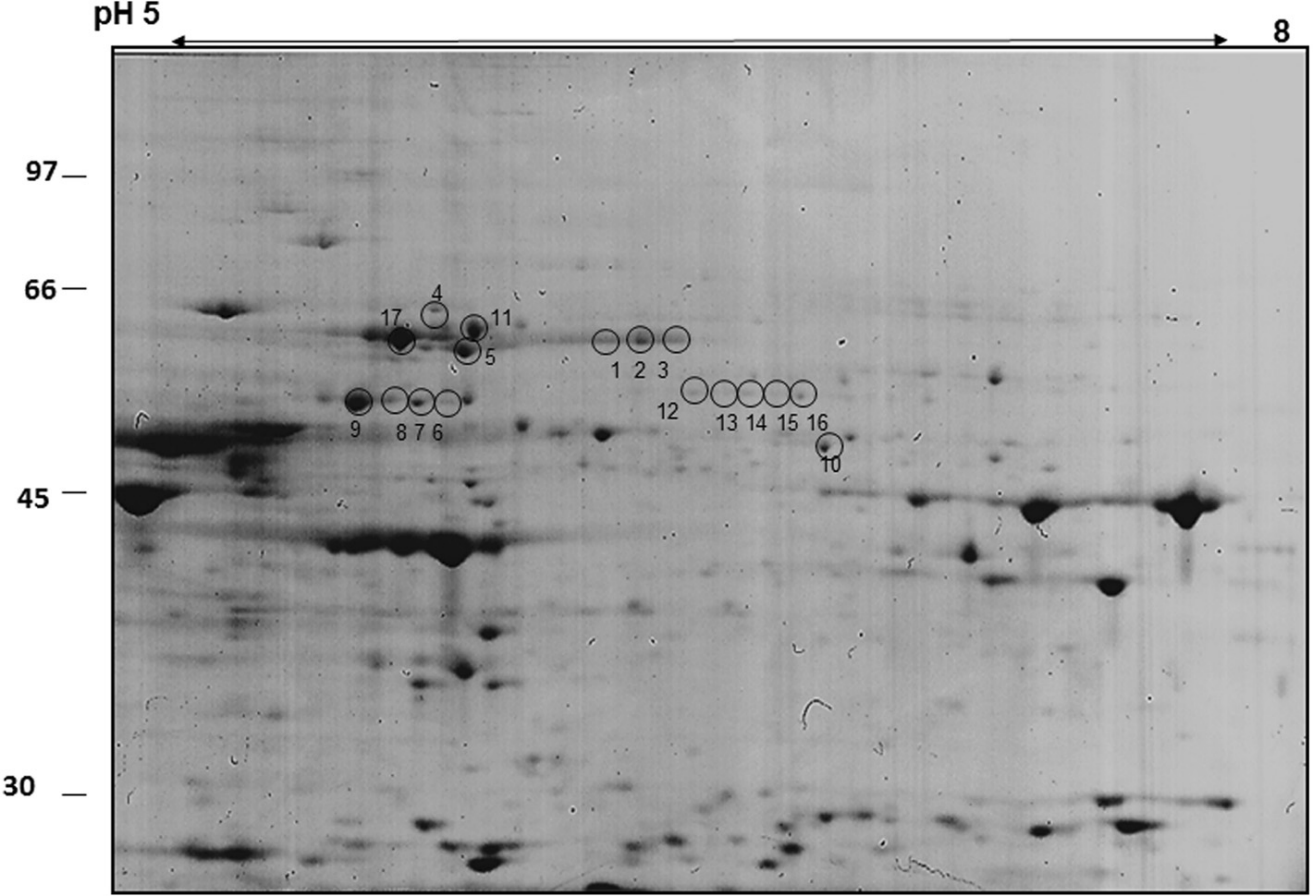
**Representative proteome profile of human retinal proteins separated by 2-D gel electrophoresis in the pH range 5–8 and stained with Coomassie Brilliant blue.** Molecular weight of standards is shown on the left and pH range on the top. Circles with numbers indicate the excised protein spots identified as heat shock proteins (HSP) or collapsin response mediator proteins (CRMP). The identified proteins are listed in Table 1.

Recognizing that one band on the 1-D gel often represents more than one protein we further focused on the identification of immunoreactive proteins by 2-D-Western blotting based on the autoantibody recognition pattern for 10 randomly selected sera that previously have shown binding to a ~62-kDa protein. Figure 2A shows immunoblots for 10 serum autoantibodies that bind to different retinal proteins within the molecular range of 60 to 70-kDa (boxed). These 10 sera were selected to be further analyzed by 2-D western blotting (shown in Figure 3). Specific anti-hsp60 and hsp70 were used as positive controls and no primary antibodies as a negative control. Figure 2B shows a representative 2-D western blot for serum#3 (Figure 2A) immunoreacting with 3 molecular forms of 62-kDa protein. Figure 4 shows a representative Coomassie blue stained gel with 17 marked proteins of interest located at the 60 to 70-kDa range and the p*I* range of 5.5 to 6.5 and immunoreacting with patients’ sera. Based on immunoreactivity with sera the reactive spots were excised from 2-D gel and identified by in-gel digestion and mass spectrometry. Table 1 present 17 successfully identified spots that belonged to the stress protein family, including mitochondrial heat shock protein 60-kDa, heat shock 70-kDa protein 1, heat shock cognate 71-kDa protein, heat shock 70-kDa protein 1 L, mitochondrial stress-70 protein, heat shock-related 70-kDa protein 2, and spots related to collapsin response mediator proteins (CRMPs; dihydropyrimidinase-related proteins), including CRMP-2 (DPYL2_HUMAN) and CRMP-3 (DPYL3_HUMAN). Multiple isoforms visible on the gel of these 6 heat shock proteins and two CRMP proteins could be caused by post-translational modifications. Two-D western blots using 8 of the 10 sera (presented in Figure 2) were used to localize which of the proteins in the 60–70 kDa and pI 5–6.5 range were potentially immunoreactive (Figure 3B-I). Among heat shock proteins, the mitochondrial 60-kDa hsp was most often recognized by AAbs. Similarly, immunoreactive spots migrated in similar positions to CRMP-2/3. The presence of multiple CRMP spots with the same molecular weight is not surprising since CRMPs are highly phosphorylated proteins [12].

**Table 1:** Identification of human retinal proteins found in spots cut out of a Coomassie-stained 2D gel by mass spectrometry

**Spot number**	**Major protein(s)**	**Accession**	**Spectral counts**
1	Heat shock cognate 71 kDa protein	HSP7C_HUMAN	89
2	Heat shock cognate 71 kDa protein	HSP7C_HUMAN	59
	Heat shock 70 kDa protein 1A/1B	HSP71_HUMAN	30
3	Stress-70 protein, mitochondrial	GRP75_HUMAN	29
	Heat shock cognate 71 kDa protein	HSP7C_HUMAN	22
4	Heat shock cognate 71 kDa protein	HSP7C_HUMAN	14
	60 kDa heat shock protein, mitochondrial	CH60_HUMAN	8
5	Alpha-internexin	AINX_HUMAN	10
	Vimentin	VIME_HUMAN	7
	Dihydropyrimidinase-related protein 2	DPYL2_HUMAN	6
6	60 kDa heat shock protein, mitochondrial	CH60_HUMAN	40
7	60 kDa heat shock protein, mitochondrial	NUCB1_HUMAN	18
	Alpha-internexin	AINX_HUMAN	11
	Pyruvate kinase isozymes M1/M2	KPYM_HUMAN	8
8	Nucleobindin-1	NUCB1_HUMAN	11
	Alpha-internexin	AINX_HUMAN	10
	Alpha-1-antitrypsin	A1AT_HUMAN	8
	Rab GDP dissociation inhibitor alpha	GDIA_HUMAN	7
	Protein kinase C and casein kinase	PACN1_HUMAN	6
	substrate in neurons protein 1		
9	Rab GDP dissociation inhibitor alpha	GDIA_HUMAN	8
	Nucleobindin-1	NUCB1_HUMAN	7
	Pyruvate kinase isozymes M1/M2	KPYM_HUMAN	6
	Alpha-1-antitrypsin	A1AT_HUMAN	5
10	Succinyl-CoA:3-ketoacid coenzyme A transferase 1	SCOT1_HUMAN	17
11	Stress-70 protein, mitochondrial	GRP75_HUMAN	81
12	Dihydropyrimidinase-related protein 2	DPYL2_HUMAN	57
13	Dihydropyrimidinase-related protein 3	DPYL3_HUMAN	28
14	Dihydropyrimidinase-related protein 3	DPYL3_HUMAN	21
15	Dihydropyrimidinase-related protein 3	DPYL3_HUMAN	20
16	Dihydropyrimidinase-related protein 3	DPYL3_HUMAN	14
17	60 kDa heat shock protein, mitochondrial	CH60_HUMAN	60

Spots were cut out of a Coomassie-stained 2D gel of human retinal proteins in a molecular weight range from approximately 60–70 kDa and pI of 5.0-6.5 and subjected to in-gel digestion with trypsin. See Figure 3 for spot numbers.

CRMPs are a family of neuronal cytoplasmic proteins present in adult central and peripheral neurons and they define axon/dendrite fate and axonal growth of neurons through protein-protein interactions. They are also present in cancer cells, including small cell lung carcinomas and AAbs against CRMP-2 [13], CRMP-5 [14], and CRMP-3-4 [15] were reported in association with cancer. Remarkably, our CAR patients with anti-CRMP-2 AAbs were diagnosed with melanoma, breast cancer, and lymphoma (Figure 3G, H, I).

**Figure 5:**
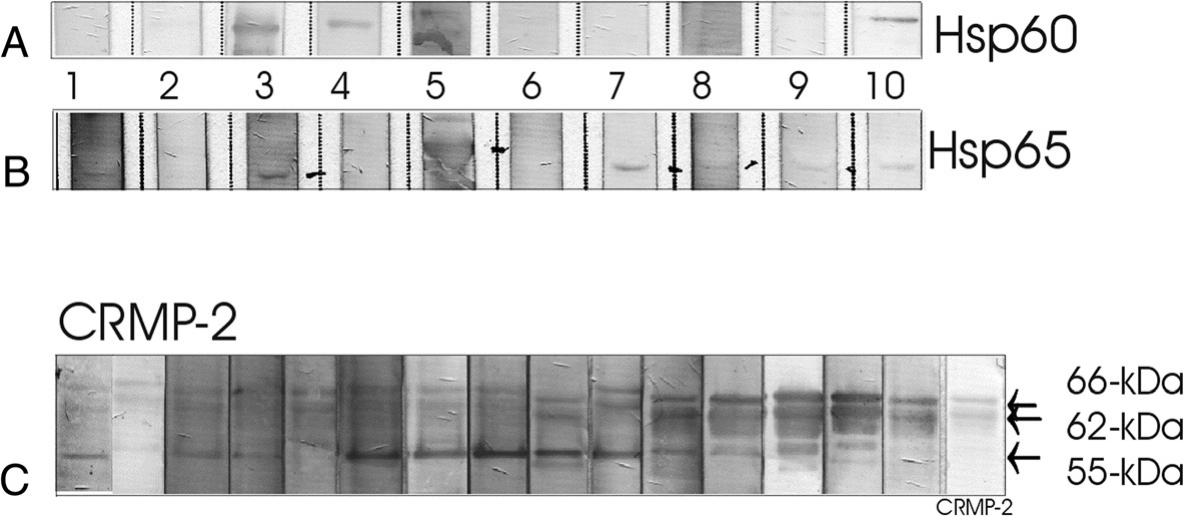
**Confirmation western blotting using purified proteins for representative patients’ sera.** Two hsp60s were used with sera presented in Figure 2. **(A)** Recombinant human hsp60 and **(B)** recombinant M. bovis Hsp65 (native) proteins (member of the hsp60 family of heat shock proteins); Note that some AAbs recognized only hsp65. **(C)** Recombinant CRMP-2 incubated with 15 patients’ sera that were suspected to react with CRMP-2. Patients’ AAbs bound to different molecular forms of CRMP-2, suggesting they recognized different epitopes. We used specific anti-human antibodies against CRMP-2 as positive control.

The results were validated for 15 sera by 1-D western blotting (Figure 5) using purified 60-kDa and 65-kDa heat shock proteins and CRMP-2 protein. Figure 5C demonstrated that sera were binding to different isoforms of CRMP-2. Unfortunately other purified CRMP proteins are not available for testing. Since the purified recombinant CRMP-2 preparation contained several lower molecular weight bands that were also immunoreactive, the results suggested that some AAbs bound more tightly to truncated forms of CRMP-2. The immunoreactivity of many of the sera with purified hsp60, hsp65, and CRMP-2 provided additional evidence supporting the putative identifications of hsp60 and CRMP-2 as immunoreactive proteins on the 2-D western blots.

**Figure 6:**
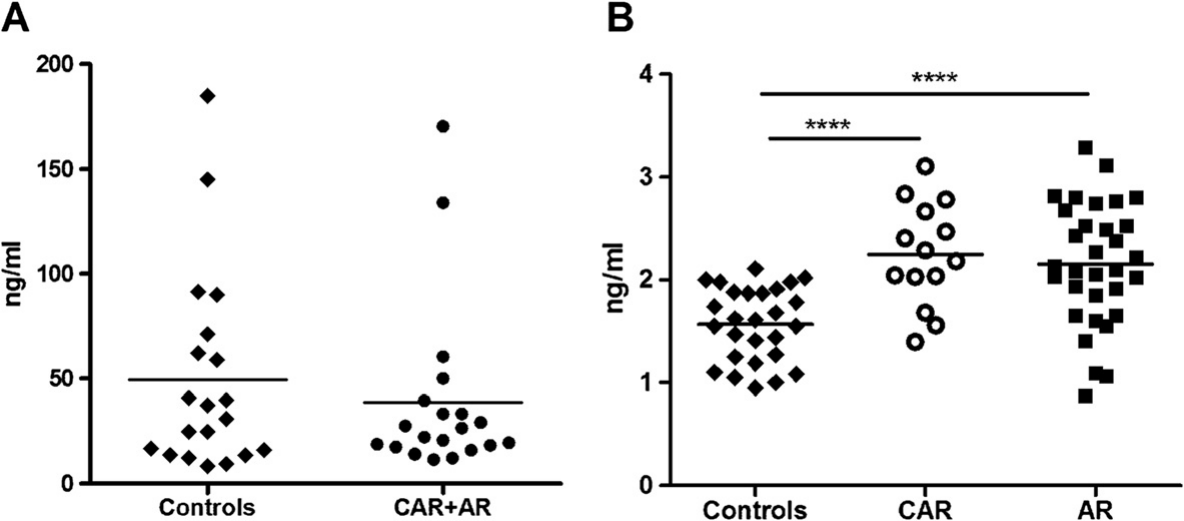
**Quantification of serum hsp60 and anti-hsp60 antibodies in patients with cancer associated retinopathy (CAR), autoimmune retinopathy (AR), and healthy subjects as controls. (A)** Levels of circulating serum hsp60 in 20 AR patients and 20 controls; the mean concentration of hsp60 49.6 ± 9 ng · mL^−1^ in control sera and 38.8 ± 10 ng · mL^−1^ in AR sera; **(B)** Serum levels of human anti-hsp60 IgG/A/M in the cohort of 27 controls, 14 CAR, and 32 AR patients were measured by ELISA; Anti-hsp60 AAbs were significantly elevated levels of in CAR (2.24 ±0.13 ng · mL^−1^) and AR sera (2.15 ± 0.10 ng · mL^−1^) compared to healthy subjects (1.56 ± 0.06 ng · mL^−1^; One-way ANOVA, p < 0.0001). The line represents the mean level for tested groups in **A** and **B**.

Next, we investigated the level of hsp60 and anti-hsp60 antibodies in the serum of AR patients and healthy subject controls. Elevated levels of hsp60 were associated with stress and cancer, e.g. colorectal cancer patients [16,17]. Hsp60 is mainly located in the mitochondria but can change its location to the cytosol and cell surface and then becomes available [18]. We performed 2 quantitative assays; initially, we examined the level of circulating hsp60 in the serum and then we determined serum anti-hsp60 antibody levels. The quantification of hsp60 in 20 AR patients and 20 healthy individuals sera showed the mean concentration hsp60 of 49.6 ± 9 ng · mL^−1^ in control sera and 38.8 ± 10 ng · mL^−1^ in AR sera; however, there was no statistical difference between groups in serum hsp60 levels (Figure 6A). In contrast, there was a significant difference in serum AAb levels between control, AR and CAR groups. Figure 6B shows the total serum IgG/A/M against human hsp60 in 14 CAR patients, 32 AR patients, and 27 healthy subjects. There were significantly elevated levels of anti-hsp60 AAbs in CAR (2.24 ±0.13 ng · mL^−1^) and AR sera (2.15 ± 0.10 ng · mL^−1^) compared to healthy subjects (1.56 ± 0.06 ng · mL^−1^; One-way ANOVA, p < 0.0001), suggesting these antibodies can be a biomarker for autoimmune retinopathy.

## Discussion

Anti-retinal autoantibodies of different specificities have been liked to AR and CAR. Our studies identified often targeted retinal proteins in the range 60-70-kDa as putative heat shock proteins and CRMP proteins and these specific AAbs were present alone or in association with other antibodies. It is not undetermined why patients with retinopathy possess AAbs that targeted hsps. Although the role of anti-hsp antibodies in retinopathy is unknown experimental findings from animal studies support their pathogenic role. The hsp70 family of proteins is best known among chaperon proteins for protection against stress-induced protein degeneration [19,20]. Blocking the function of hsps by specific AAbs is likely to remove protective effects of such proteins in the cell. Ohguro et al. showed serum AAbs against recoverin and heat shock cognate protein 70 (hsc70) concurrently present in four Japanese CAR patients, suggesting that anti-hsc70 AAbs might intensified pathogenic effects of anti-recoverin AAbs on the retina during the autoimmune process [21]. In our patient’s cohort, 37 out of 819 sera revealed anti-recoverin AAbs, and among them, about 30% anti-recoverin-positive sera had additional AAbs against 60-70-kDa retinal proteins. Out of 15 CAR patients with anti-recoverin AAbs eight had lung cancer but only three of those patients had additional anti-67-kDa AAbs. Because such a low incidence of co-existence of anti-recoverin/anti-67-kDa in our cohort of Caucasian patients we speculated, that perhaps, the generation of anti-67-kDa antibody is genetically restricted and the ethnic background may predispose individuals to the generation of such AAbs. More patients of different ethnicity must be examined to determine whether this hypothesis holds up.

Also, AAbs against a 70-kDa hsp were found in serum of patients with glaucoma and were increased in aqueous humor of those patients compared with the non-glaucoma group, which supports an autoimmune involvement in the pathogenic process in glaucoma [22]. Furthermore, immunization of Lewis rats with hsp27 and hsp60 induced the retinal ganglion cell degeneration and axonal loss in a pattern similar to human glaucoma, including topographic cell loss in this glaucoma rat model [23]. In animal studies of CAR in Lewis rats, the CAR-like retinal dysfunction was more pronounced when anti-recoverin antibodies were co-administrated with antibodies against hsp70 by intravitreal injection [21]. Therefore, it is possible that in susceptible patients, autoimmunity to hsp plays a role in augmenting the effects of coexisting pathogenic AAbs against other retinal proteins, such as recoverin or α-enolase.

Hsp60 is an immunodominant antigenic component of different common pathogens, thus there is a consensus that hsp60-specific antibodies may be developed in response to infections as a defense mechanism against pathogens. In fact, our patients AAbs bound better to hsp65, which is a microbial form of hsp60 (Figure 5). A high degree of antigenic homology (47%) between microbial (bacterial and parasitic) and human hsp60 could possibly cause infection-induced autoimmunity [20]. The homology between bacterial and human hsp could also explain the presence of these AAbs in the serum of healthy individuals [24,25]. Therefore, anti-hsp60 antibodies in the serum of healthy individuals may represent the natural autoantibody repertoire, whose formation might be in response to common infections acquired throughout life. Molecular mimicry has been suggested as a mechanism for the formation of AAbs and autoreactive T cells against hsp that were upregulated during stress [26]. Moreover, hsp also *share homology* with retinal proteins, such as IRBP which could amplify pathogenicity of anti-retinal AAbs/T cells throughout the disease process [27,28]. Taken together, the increased levels of anti-hsp AAbs in patients with AR suggests that elevated anti-hsp immune responses may have a contributory role in autoimmune retinal degeneration and needs to be further investigated.

CRMP-1-4 exhibit 68-75% sequence identity with one another but the sequence composition of CRMP-5 differs significantly from CRMP-1-4 and shows only about 50% homology [29]. To verify the reactivity with CRMP-2 we used human recombinant CRMP-2 protein on the blot. Figure 3C shows 15 serum samples that recognized different CRMP-2 molecular forms. The 64- and 66-kDa bands of CRMP-2 are two phosphorylated isoforms of 62-kDa CRMP-2 [30] and both isoforms were recognized by AAbs on the blots. Moreover, seven sera strongly bound to the 55-kDa breakdown product (BDP) of CRMP-2 [31,32], suggesting that these AAbs recognized different fine epitopes on the protein. AAbs against CRMP 2 and 3 have not been described in retinopathy before so they could be considered as novel biomarkers for the disease and their pathogenic properties will be further investigated.

## Conclusion

In conclusion, different anti-retinal antibodies frequently co-exist in a single patient, creating antibody-arrays related to the syndrome. We believe that prompt identification of antigenic targets may lead to better diagnosis of AR and associated cancer and understating the causative role of AAbs in retinal pathology. Anti-hsp60 AAbs could be initiated in response to microbial and oncological antigens that also cross-react with antigens in the retina. Because of the frequent association of anti-hsp antibodies with other anti-retinal antibodies in the same patient, it is possible that they augment the pathogenic effects on the retina, leading to cell death and retinal degeneration. An additional research is also needed to investigate the relationship between circulating anti-hsp60 in the progression of AR. Regardless of their role in development of AR these AAbs can serve as useful clinical predictors of autoimmune retinal degeneration.

## Competing interests

The authors declare that they have no competing interests.

## Authors’ contributions

All authors made contribution to the manuscript. GA contributed to the study conception and design, providing funding for materials, interpreting data, drafting and critically revised the manuscript. LD contributed to design, providing funding for materials, interpreting data, and critically revised the manuscript. RB and LB designed and preformed experiments, collected and analyzed data. All authors read and approved the final manuscript.

## Pre-publication history

The pre-publication history for this paper can be accessed here:

http://www.biomedcentral.com/1471-2415/13/48/prepub
